# Shengxue Busui Decoction activates the PI3K/Akt and VEGF pathways, enhancing vascular function and inhibiting osteocyte apoptosis to combat steroid-induced femoral head necrosis

**DOI:** 10.3389/fphar.2024.1506594

**Published:** 2025-01-24

**Authors:** Manting Liu, Jiexiang Ye, Runtian Wu, Dongqiang Luo, Tao Huang, Dandan Dai, Kexin Wang, Yanping Du, Junwen Ou

**Affiliations:** ^1^ Clifford Hospital, Guangzhou University of Chinese Medicine, Guangzhou, China; ^2^ The Eighth Clinical Medical College of Guangzhou University of Chinese Medicine, Foshan, China; ^3^ Guangzhou Hospital of Integrated Traditional and Western Medicine Affiliated to Guangzhou University of Chinese Medicine, Guangzhou, China; ^4^ The Second Clinical Medical College of Guangzhou University of Chinese Medicine, Guangzhou, China; ^5^ The First Clinical Medical College of Guangzhou University of Chinese Medicine, Guangzhou, Guangdong, China

**Keywords:** osteocyte apoptosis, steroid-induced femoral head necrosis, network pharmacology, PI3K/Akt pathway, VEGF pathway

## Abstract

**Introduction:**

Steroid-induced osteonecrosis of the femoral head (SONFH) is a debilitating condition with no specific treatment. Inhibiting osteocyte apoptosis may be a promising therapeutic approach. Shengxue Busui Decoction (SBD) has shown protective effects against SONFH, but its mechanisms are not fully understood. This study aims to investigate the effects of SBD on SONFH in rats, identifying its key active components and regulatory mechanisms using network pharmacology, bioinformatics, machine learning, and experimental validation.

**Methods:**

Key active components and disease targets of SBD were identified through network pharmacology and bioinformatics. GO/KEGG enrichment and ssGSEA analyses were performed to identify critical pathways. Cytoscape and machine learning (SVM) were used for target prediction and molecular docking validation. A dexamethasone (Dex)-induced SONFH rat model was established, and SBD was administered for 60 days. Histological changes were assessed via HE staining, osteoclast activity through TRAP staining, apoptosis levels with TUNEL assays, and vascular function through hematological tests. ELISA was used to measure ALP and OCN levels. *In vitro*, Dex-induced osteoblast apoptosis in MC3T3-E1 cells was examined to assess SBD’s effect on osteoblast proliferation, apoptosis, and signaling. Western blotting analyzed Caspase-9, Caspase-3, Bax, Bcl-2, and pathway-related proteins. ALP and Alizarin Red staining evaluated osteoblast differentiation and mineralization.

**Results:**

Network pharmacology identified curcumin, berberine, and diosgenin as key active components of SBD, with the PI3K/Akt and VEGFR pathways as critical targets, and RAF1, FOXO3, and BRAF as hub genes. *In vivo*, SBD intervention significantly reduced bone structural damage and apoptosis, decreasing the rate of empty bone lacunae. SBD also increased osteogenic markers ALP and OCN in SONFH rats. *In vitro*, SBD inhibited osteoblast apoptosis, promoted PI3K/Akt and VEGF pathway expression, and enhanced osteoblast differentiation and mineralization.

**Conclusion:**

This study integrates network pharmacology with experimental validation, showing that SBD protects against SONFH by inhibiting osteoblast apoptosis via PI3K/Akt and VEGFR pathways. SBD promotes osteoblast differentiation and mineralization, improving bone structure and vascular function. Curcumin, berberine, and diosgenin are likely key contributors to these effects, highlighting SBD as a potential therapeutic strategy for SONFH.

## 1 Introduction

Osteonecrosis of the femoral head (ONFH) is a challenging orthopedic condition marked by hip pain and dysfunction, leading to high disability rates. Over 10,000 new cases are reported annually in the U.S. (Mont et al., 2015), accounting for 10% of hip arthroplasties, with 120,000 cases in China over 8 years (Zhao et al., 2015). Steroid use is a significant risk factor, particularly for steroid-induced ONFH (SONFH) in young and middle-aged adults, with incidence rising due to the COVID-19 pandemic (Zhang et al., 2021). The pathogenesis of SONFH is complex and not fully understood (Luo et al., 2024). Current Western medical treatments for SONFH, such as core decompression, bone grafting, and osteotomy, are limited by delays, high costs, significant damage, and numerous complications, severely affecting patients’ quality of life (Wu et al., 2015). Traditional Chinese Medicine (TCM) offers effective alternatives with fewer side effects (Jiang et al., 2021; Liu et al., 2016). TCM categorizes SONFH under “bone erosion” and attributes it to liver and kidney deficiencies. Chen (2012) reported a 96.1% success rate utilizing SBD combined with minimally invasive decompression in treating SONFH, indicating a promising future for TCM. This study aims to identify SBD’s active ingredients and targets using network pharmacology, bioinformatics, and machine learning, with molecular docking and animal experiments validating findings for SONFH treatment development.

## 2 Materials and methods

### 2.1 Screening of SONFH-related targets

Using the keyword “steroid induced osteonecrosis of femoral head,” we retrieved the SONFH-related dataset GSE123568 from the GEO database (https://www.ncbi.nlm.nih.gov/geo) on the GPL15207 platform, Affymetrix Human Gene Expression Array.GSE123568 contains 40 SONFH samples and 30 healthy samples from peripheral serum. The dataset was log2 normalized utilizing R,differential analysis was conducted utilizing the limma package (Ritchie et al., 2015).Differential genes with |logFC ≥ 0.5| and *P* < 0.05 were obtained, and differential gene volcano plots were drawn.

### 2.2 Screening of active compounds and related targets of SBD

Using the TCMSP database (https://tcmsp-e.com/tcmsp.php), we searched for the active components and targets of SBD with keywords “*Rehmannia*”, “*White Peony*”, “*Ligusticum*”, “*Astragalus*”, “*Achyranthes*”, “*Eucommia*”, “*Safflower*”, “*Acanthopanax*”, “*Angelica*”, and “*Dipsacus*”. Criteria were set with oral bioavailability (OB) ≥30% and drug-likeness (DL) ≥0.18. Additional components and targets were identified utilizing the BATMAN-TCM database. After removing duplicates, results were imported into the UniProt database (https://www.uniprot.org/) with the species set to “*Homo Sapiens*” and corrected the target proteins to their official gene symbols.

### 2.3 Construction of protein-protein interaction networks

The standard targets between SONFH-related and SBD drug targets were identified as potential therapeutic targets for SBD against SONFH and visualized with a Venn diagram. These targets were then imported into the STRING 12.0 platform with the species set to “*Homo sapiens*” and a minimum required interaction score of “Highest Confidence (0.900)” utilizing default parameters to construct the PPI network.

### 2.4 Enrichment analysis

The “clusterProfiler” and “org.Hs.eg.db”packages were used to perform GO biological process enrichment and KEGG pathway annotation for PPI network nodes. GO enrichment included MF (molecular function), CC (cellular component), and BP (biological process). Pathways with P.adj<0.05 were considered key biological processes for SBD’s action against SONFH.

### 2.5 ssGSEA analysis

To further validate the role of enriched pathways in SONFH, the “c2.cp.kegg.symbols.gmt” file was obtained from the GSEA database (https://www.gsea-msigdb.org/gsea/index.jsp), which includes KEGG pathway target information. Utilizing the “GSVA” package, ssGSEA analysis was conducted on SONFH and control groups to compare enrichment score differences. This identified biological processes related to SONFH onset. The common pathways enriched in SBD treatment and SONFH were identified as critical pathways for disease onset and treatment.

### 2.6 Identification of key targets

The PPI network was analyzed using Cytoscape 3.7.1 to calculate node degree and betweenness centrality, identifying target importance and roles in information transfer. The MCODE algorithm detected highly interconnected modules, selecting the subnetwork with the highest correlation score. Key targets and main active compounds were identified based on node degree, with higher degrees indicating greater network significance.

### 2.7 Identification of hub genes utilizing machine learning

This study delved into four classifier models to identify hub genes associated with SONFH and develop an ultimate predictive model. By using disease status as a binary outcome and tuning SVM, RF, and XGBoost with extensive hyperparameter adjustments, the best classifier was selected based on AUC values. Cross-validation and grid search via the caret package optimized parameters (Kuhn, 2008), while the DALEX package (Biecek, 2018) identified the top three hub genes through five-fold validation. The optimal model, reconstructed with these hub genes and the best classifier, was then evaluated for performance and net benefit using clinical decision curves (Luo et al., 2024).

### 2.8 Molecular docking

The most significant active compounds were selected for molecular docking with RAF1, FOXO3, and BRAF. The PDB files for these core target proteins were downloaded from the Protein Data Bank (PDB) (https://www.rcsb.org/), and the criteria included human origin, resolution, and presence of original ligands. The mol2 files for active compounds were obtained from the TCMSP database. PyMOL 2.6.0 was used to remove water and small ligands from target proteins, and AutoDockTools 1.5.7 was employed to add hydrogens for docking. The docking results were visualized with PyMOL to evaluate binding energies between active compounds and hub genes. Generally, lower binding energy indicates more stable interactions.

### 2.9 Cell culture

Mouse pre-osteoblasts (MC3T3-E1), obtained from the China Center for Type Culture Collection (ATCC No. CRL-2593), were cultured in DMEM supplemented with 10% fetal bovine serum (FBS, HyClone, United States) at 37°C in a 5% CO_2_ atmosphere.

### 2.10 ALP staining

MC3T3-E1 cells were seeded in 24-well plates at a density of 1 × 10^5^ cells per well. Following specified treatment and a 24-h incubation, the medium was removed, and 200 μL of 1% Triton X-100 solution was added. Cells were lysed at 4°C for 1 hour. Subsequently, Alkaline Phosphatase (ALP) activity and protein content were determined and calculated using an ALP assay kit (ml003360, Elisa, China).

### 2.11 Alizarin Red staining

MC3T3-E1 cells were seeded in 24-well plates at 1 × 10^5^ cells per well and cultured in osteogenic differentiation medium at 37°C with 5% CO_2_. Cells were treated according to experimental groups. On day 15, cells were fixed with 95% ethanol, stained with 0.1% Alizarin Red for 30 min, rinsed, dried, and subsequently observed and photographed under a microscope.

### 2.12 Animal preparation and medicinal serum preparation

Male Wistar rats, aged 8–10 weeks and weighing 180–200 g, were obtained from the Guangzhou Riegel Biotechnology Co., Ltd. Animal Center. They were housed in a pathogen-free environment at a temperature of 22°C ± 2°C and a relative humidity of 50% ± 5%, with a 12-h light/dark cycle. The rats underwent a 1-week acclimatization period before the experiments, with free access to food and water. The Ethics Committee of Guangzhou Riegel Biotechnology Co., Ltd. approved all experimental procedures.

The SBD formula (*Rehmannia* 30 g, *Ligusticum* 15 g, *Astragalus* 20 g, *Eucommia* 20 g, *White Peony* 15 g, *Angelica* 15 g, *Acanthopanax* 15 g, *Achyranthes* 25 g, *Safflower* 10 g, *Dipsacus* 25 g) was provided by the Pharmacy Department of Guangdong Qifu Hospital. All herbs were soaked in five times their volume of water for 30 min and then boiled for 30 min. The mixture was filtered through four layers of gauze, and the filtrate was boiled again with four times the volume of water. The extract was concentrated utilizing a rotary evaporator and stored at 4°C for future use.

Rats were randomly divided into five groups: control (n = 5), model (n = 5), low-dose SBD (5.25 mg/kg, n = 5), medium-dose SBD (10.50 mg/kg, n = 5), and high-dose SBD (21.00 mg/kg, n = 5). The model and SBD groups received intramuscular injections of dexamethasone at 50 mg/kg twice weekly for 6 weeks to induce femoral head necrosis. The dosage conversion formula for SBD is defined as Dm = Dh/W * F (Ren et al., 2019), where Dm denotes the dosage administered to rats, Dh is the clinical dosage for humans, W represents the average adult body weight (assumed to be 60 kg), and F is the conversion factor between rat and human dosages, set at 6.3. Consequently, the standard dosage of this medication is 19.95 g/(kg·d), with the low and high dosages determined to be 9.98 g/(kg·d) and 39.90 g/(kg·d), respectively. Following the successful establishment of the model, rats in the low, medium, and high dosage SBD groups were administered oral gavage of 9.98 g/(kg·d), 19.95 g/(kg·d), and 39.90 g/(kg·d) of SBD, respectively, over a period of 60 days. The control and model groups received equivalent saline. On the final day, animals fasted for 15 h before blood collection. One hour after the final administration, 5 mL of blood was collected from the abdominal aorta. The peripheral blood was centrifuged at 1,000 *g* for 15 min, and the supernatant was incubated at 56°C for 30 min for sterilization and inactivation. The undiluted serum was then rapidly frozen and stored at −80°C. The drug-containing serum was diluted with DMEM to concentrations of 5%, 15%, and 25%, forming the low, medium, and high-dose SBD groups, respectively.

### 2.13 HE staining

For each group, the left femoral heads of five rats were decalcified utilizing 14% EDTA at pH 7.4 in an ultrasonic decalcification machine, with daily solution changes for approximately 1 week until complete decalcification. The samples were dehydrated, embedded, and sectioned into 5 μm slices. Slides were dried in a 37°C incubator and stored at room temperature. Subsequent steps included deparaffinization, hematoxylin and eosin staining, dehydration, and sealing. After staining, slides were placed in a fume hood to remove xylene odour and scanned utilizing a digital pathology system to compare trabecular morphology. Five slides were selected for each group, and three random fields above the epiphyseal line were examined at ×200 magnification to calculate the empty lacuna rate. The empty lacuna rate was determined as the number of empty lacunae divided by the total lacunae multiplied by 100%.

### 2.14 TRAP staining and osteoclast evaluation

Prepare tartrate-resistant acid phosphatase (TRAP) incubation solution and incubate the tissue sections at 37°C for 50 min. Observe under a microscope until osteoclasts appear dark red. Rinse with distilled water, stained with Harris hematoxylin for 3–8 min, and rinse with tap water. Differentiate briefly with 1% hydrochloric acid alcohol, rinse with tap water, return to blue with 0.6% ammonia water, and rinse again. Dehydrate, clear, and mount with neutral resin. Examine under a microscope and capture images for analysis.

### 2.15 TUNEL assay for apoptosis in femoral head tissue

Apoptosis was detected on femoral head paraffin sections using a TUNEL kit. The slides were stained with 0.03% DAB for 5 minutes and subsequently counterstained with hematoxylin. Brown nuclei observed under the microscope were identified as TUNEL-positive cells. Bone tissue cells were photographed and examined using an optical microscope. The apoptosis rate was calculated using the formula: Apoptosis Rate (%) = (Number of Apoptotic Cells/Total Number of Cells) × 100%.

### 2.16 Hematological analysis

Whole blood from the abdominal aorta of each group was collected for haematological analysis. A BT-300 automated blood rheometer measured hemorheological parameters, including erythrocyte aggregation index and plasma viscosity. Coagulation parameters, such as thrombin time (TT), activated partial thromboplastin time (APTT), prothrombin time (PT), and fibrinogen (FIB), were assessed. Additionally, biochemical kits were used to evaluate serum markers of vascular endothelial function, including NO and ET-1, following the manufacturer’s protocol.

### 2.17 ELISA

Serum concentrations of ALP and osteocalcin (OCN) were measured utilizing commercial ELISA kits, following the manufacturer’s instructions. Protein levels were calculated utilizing a standard curve method.

### 2.18 Western blot

As previously described (Xue et al., 2018), osteoblasts were cultured in 6-well plates (3 × 10^5^ cells/well) and treated with Dexamethasone (Dex) for specified durations. Cells were pre-treated with varying concentrations of medicated serum for 1 h before Dex treatment. At the end of the experiment, both floating and adherent cells were collected. Proteins were extracted utilizing RIPA buffer containing PMSF and quantified with a BCA Protein Assay Kit (Beyotime Biotechnology). Total protein (20–40 μg) was separated via 12% SDS-PAGE and transferred to PVDF membranes. Membranes were blocked and incubated overnight with primary antibodies (1:1,000), followed by 2 h with secondary antibodies (1:3,000). β-actin served as the loading control. Protein bands were analyzed utilizing ImageJ. Antibodies used included those against VEGFR2, PKC, Raf1, MEK, ERK, PI3K, Akt, Caspase-9, Caspase-3, Bax, Bcl2, FOXO3, and BRAF.

### 2.19 Statistical analysis

Data are presented as mean ± SD. Statistical analysis was conducted using R version 4.3.2. Differences between the two groups were evaluated using the Wilcoxon test. *P* < 0.05 is regarded as statistically significant.

## 3 Results

### 3.1 Identification of SONFH disease targets

**FIGURE 1:**
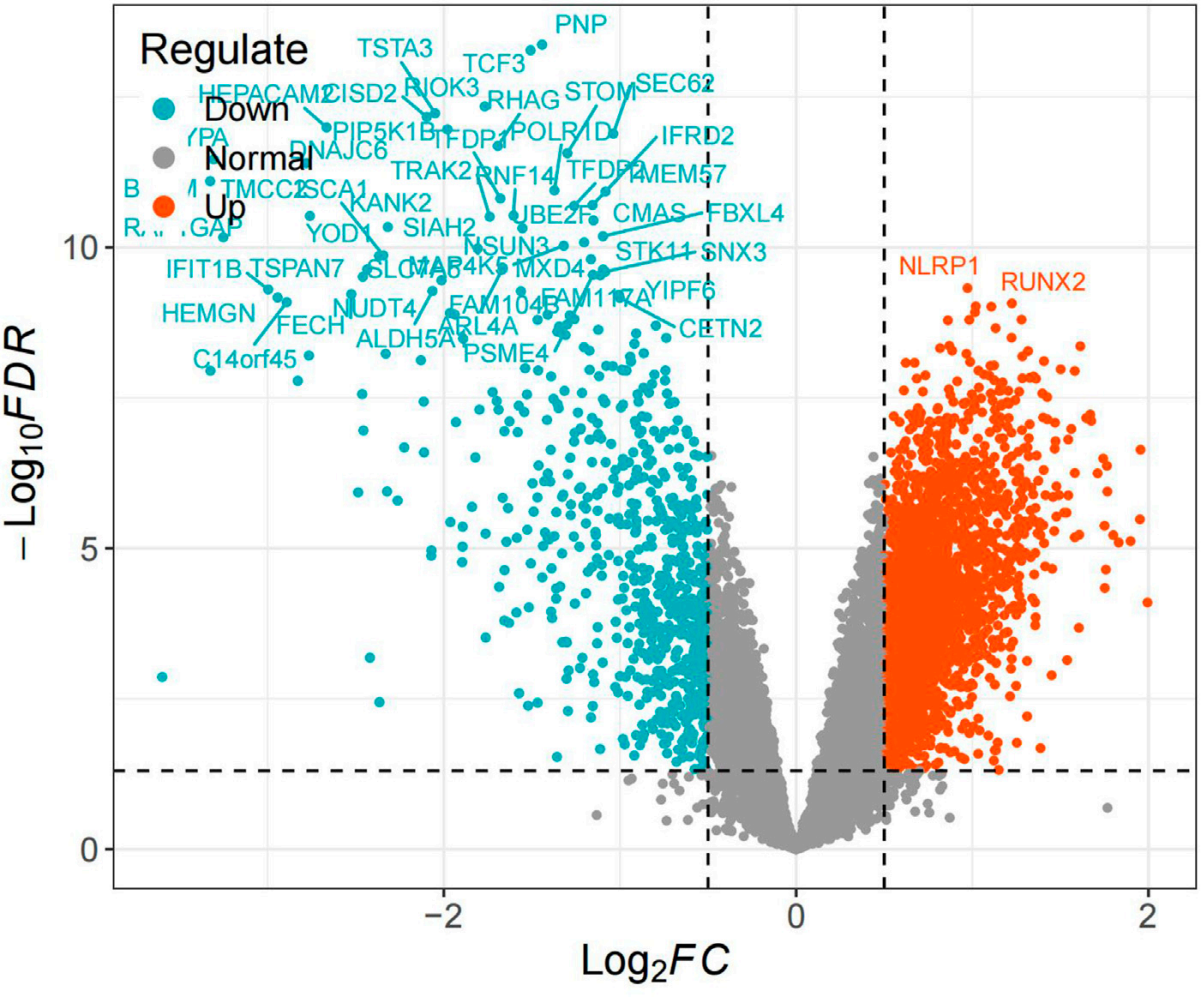
Volcano plot.

Differential expression analysis identified 3,111 differentially expressed genes (DEGs), with 2,362 upregulated and 749 downregulated (Figure 1).

### 3.2 Identification of SBD targets for Anti-SONFH action

**FIGURE 2:**
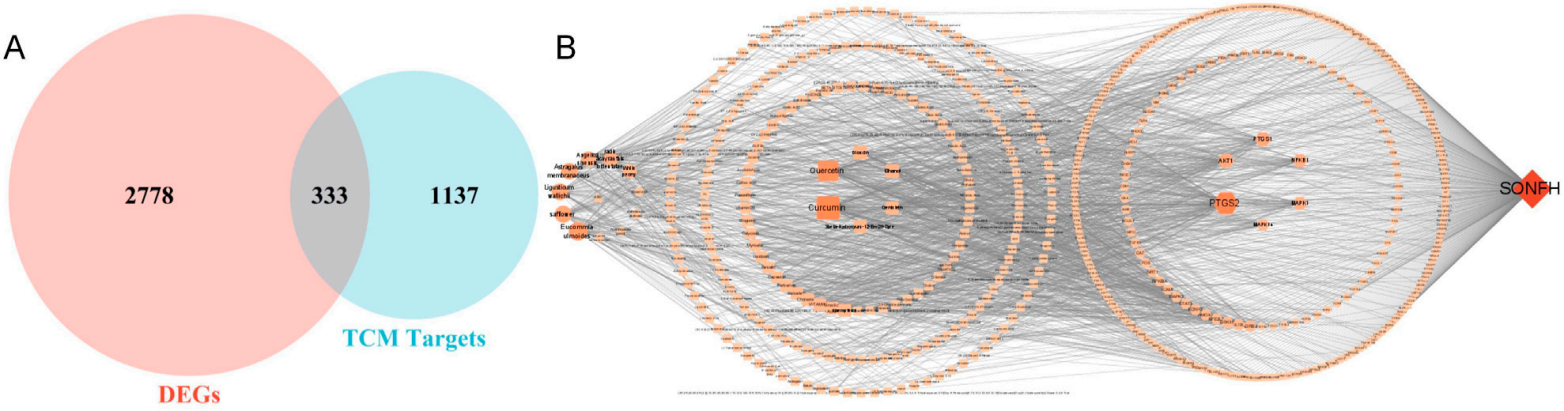
**(A)** Venn diagram; **(B)** “SBD-Herbal-Component-Target-SONFH” Network.

Active components and targets of SBD were obtained from the TCMSP and BATMAN-TCM databases. After consolidation and deduplication, 1,470 drug targets for SBD were identified. Intersection analysis revealed 333 common targets between SBD and SONFH (Figure 2A). Related herbal components were identified, and an “SBD-Herbal-Component-Target-SONFH” network was constructed utilizing Cytoscape 3.7.1. This network includes 585 nodes and 1,884 edges, highlighting SBD’s “multi-component, multi-target” nature (Figure 2B). Based on degree ranking, the primary active components identified were curcumin, berberine, and diosgenin (Table 1).

**TABLE 1:** The figure print of SBD.

Molecular number	Name	Degree	Attribution of drugs				
MOL000090	Curcumin	87	*Angelica*				
MOL000098	Quercetin	72	*Eucommia*	*Safflower*	*Astragalus*	*Achyranthes*	
MOL002441	Dioscin	33	*Achyranthes*				
MOL000776	Ethanol	32	*Angelica*	*Achyranthes*			
MOL000422	Kaempferol	28	*White Peony*	*Eucommia*	*Safflower*	*Astragalus*	*Achyranthes*
MOL000511	3beta-Hydroxyurs-12-En-28-Syre	28	*Eucommia*	*Dipsacus*			
MOL000480	Genistein	28	*Eucommia*				
MOL000511	Ursolic Acid	25	*Eucommia*				
MOL002771	VITAMIN E	25	*Safflower*				
MOL002714	Baicalein	24	*Achyranthes*	*Safflower*			
MOL000953	Cholesterol	24	*Safflower*				
MOL001454	Berberine	23	*Eucommia*	*Achyranthes*			
MOL002776	Baicalin	19	*Achyranthes*	*Safflower*			
MOL000390	Daidzein	19	*Astragalus*				
MOL002579	Capsaicin	19	*Astragalus*				

### 3.3 Construction of PPI network

**FIGURE 3:**
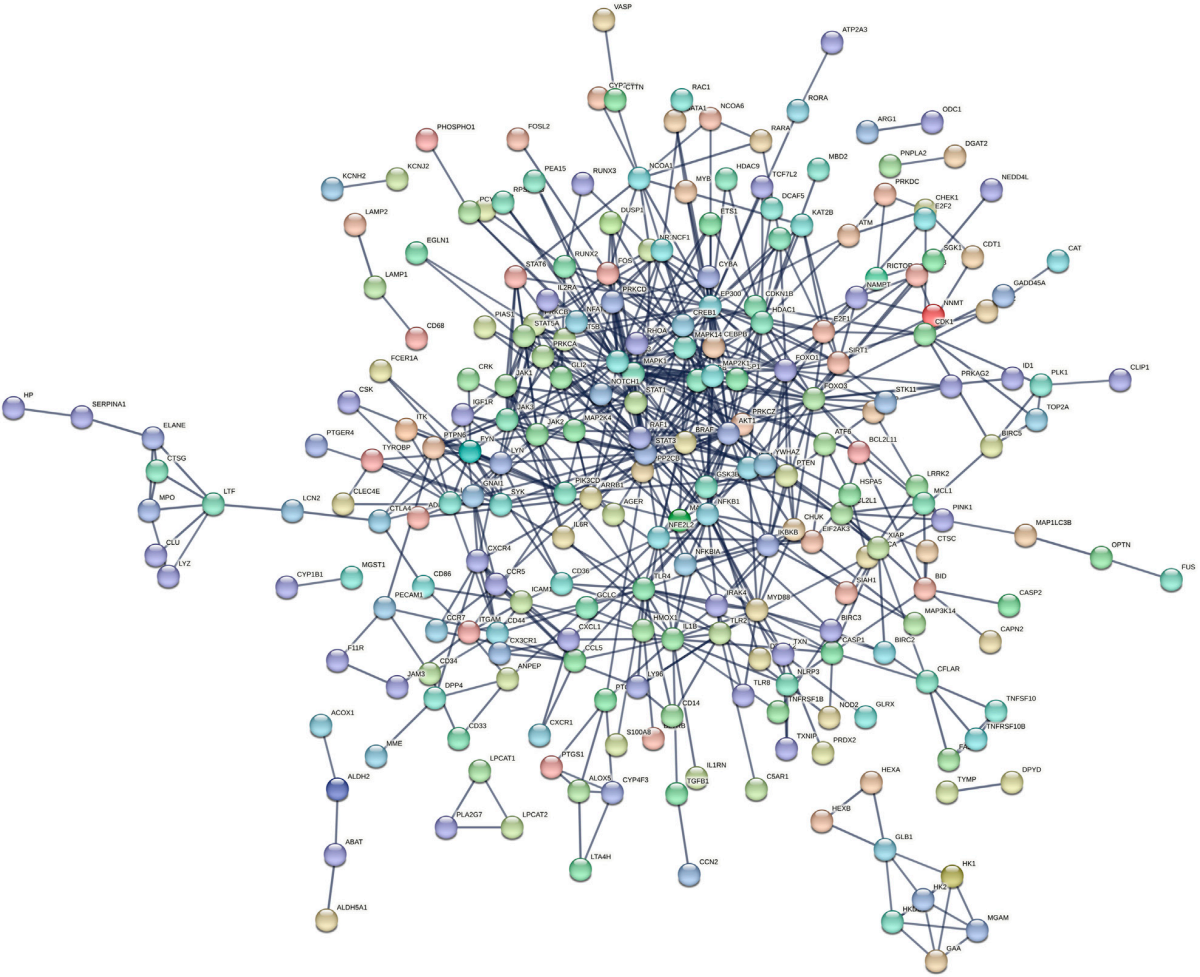
Protein–protein interaction (PPI) Network.

Three hundred thirty-three target proteins were entered into the STRING database to construct a PPI network with the highest confidence. Unconnected nodes were removed, resulting in a network with 235 nodes, demonstrating enhanced relevance (Figure 3).

### 3.4 Enrichment analysis

**FIGURE 4:**
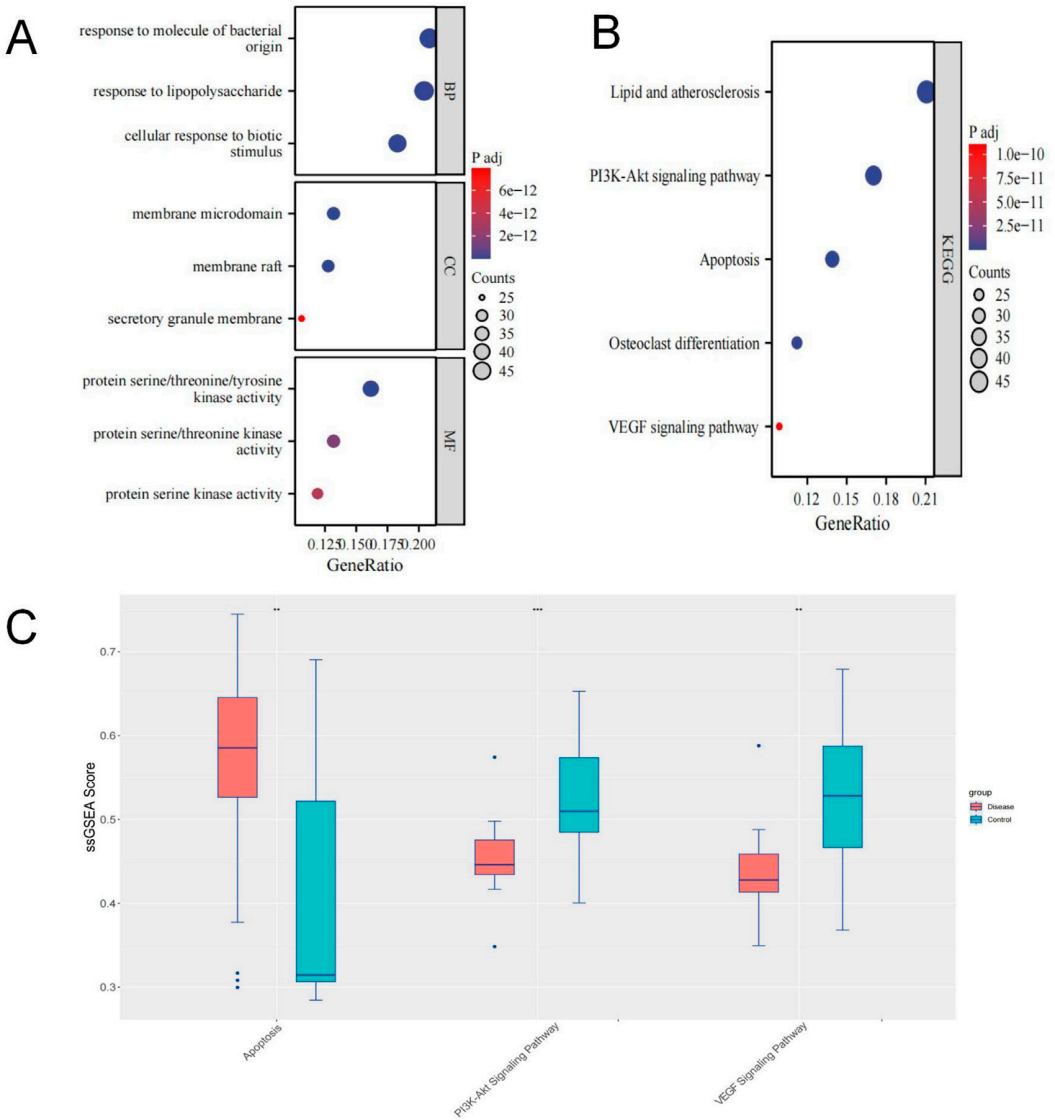
**(A, B)** GO/KEGG Enrichment Analysis; **(C)** Box plot showing differences in ssGSEA scores for Apoptosis, PI3K-Akt, and VEGF pathways.

Enrichment analysis revealed that the 235 highly relevant targets are primarily associated with biological processes such as response to molecules of bacterial origin, response to lipopolysaccharide, and cellular response to biotic stimulus. They are enriched in cellular components like membrane microdomains, membrane rafts, secretory granule membranes, and molecular functions, including protein serine/threonine/tyrosine kinase activity. Additionally, they are involved in KEGG pathways such as lipid and atherosclerosis, PI3K-Akt signalling, apoptosis, osteoclast differentiation, and VEGF signalling (Figure 4A).

We obtained target information for the PI3K-Akt signalling pathway, apoptosis, and VEGF signalling pathway from the GSEA website. Using ssGSEA, we conducted pathway enrichment scoring. Non-parametric tests revealed that the SONFH group had higher enrichment scores for apoptosis and lower scores for the PI3K-Akt and VEGF pathways (Figure 4B). These results suggest that the inhibition of PI3K-Akt and VEGF pathways and apoptosis activation may be critical mechanisms in SONFH development. BHD may improve SONFH by activating the PI3K-Akt and VEGF pathways and inhibiting apoptosis. Subsequent *in vivo* experiments will validate these findings.

### 3.5 Identification of hub genes

**FIGURE 5:**
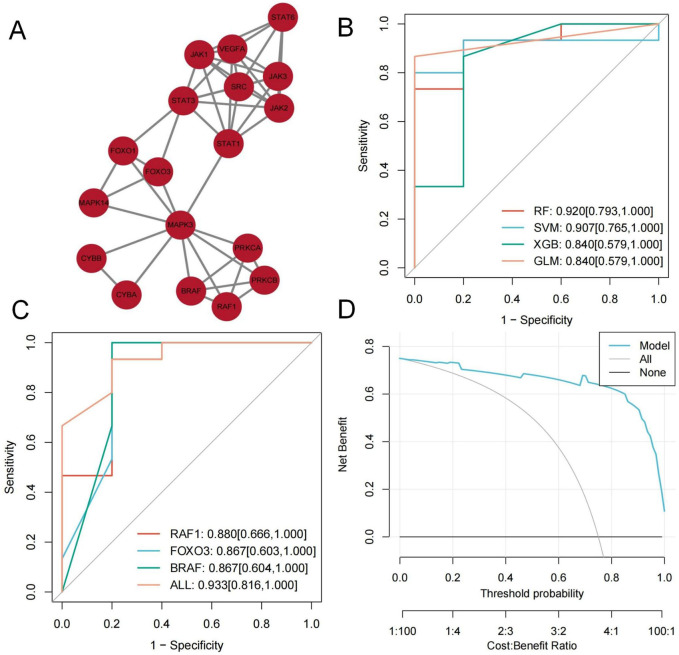
**(A)** MCODE module plot; **(B, C)** RF classifier model Graphic; **(D)** clinical decision curves Graphic.

Using the MCODE module, we extracted a cluster with a higher connectivity density (score: 5.647), consisting of 18 nodes and 48 edges, identified as key targets. These include VEGFA, JAK3, CYBB, JAK2, STAT1, BRAF, FOXO3, RAF1, PRKCB, STAT3, PRKCA, JAK1, SRC, FOXO1, STAT6, MAPK3, CYBA, and MAPK14 (Figure 5A).

We constructed various classifier models based on critical targets utilizing the training set. The RF classifier demonstrated the highest predictive performance (AUC = 0.920 [95% CI: 0.793–1.000]) (Figure 5B). As the optimal model, its feature importance was evaluated utilizing the DALEX package. The top three targets, RAF1, FOXO3, and BRAF, were identified as hub genes.

Based on the RF classifier model and hub gene features, we developed a diagnostic prediction model with excellent performance (RAF1: AUC = 0.880, 95% CI: 0.666–1.000; FOXO3: AUC = 0.867, 95% CI: 0.603–1.000; BRAF: AUC = 0.867, 95% CI: 0.604–1.000; All: AUC = 0.933, 95% CI: 0.816–1.000) (Figure 5C). Clinical decision curves indicate this model offers substantial net benefit (Figure 5D).

### 3.6 Molecular docking

**FIGURE 6:**
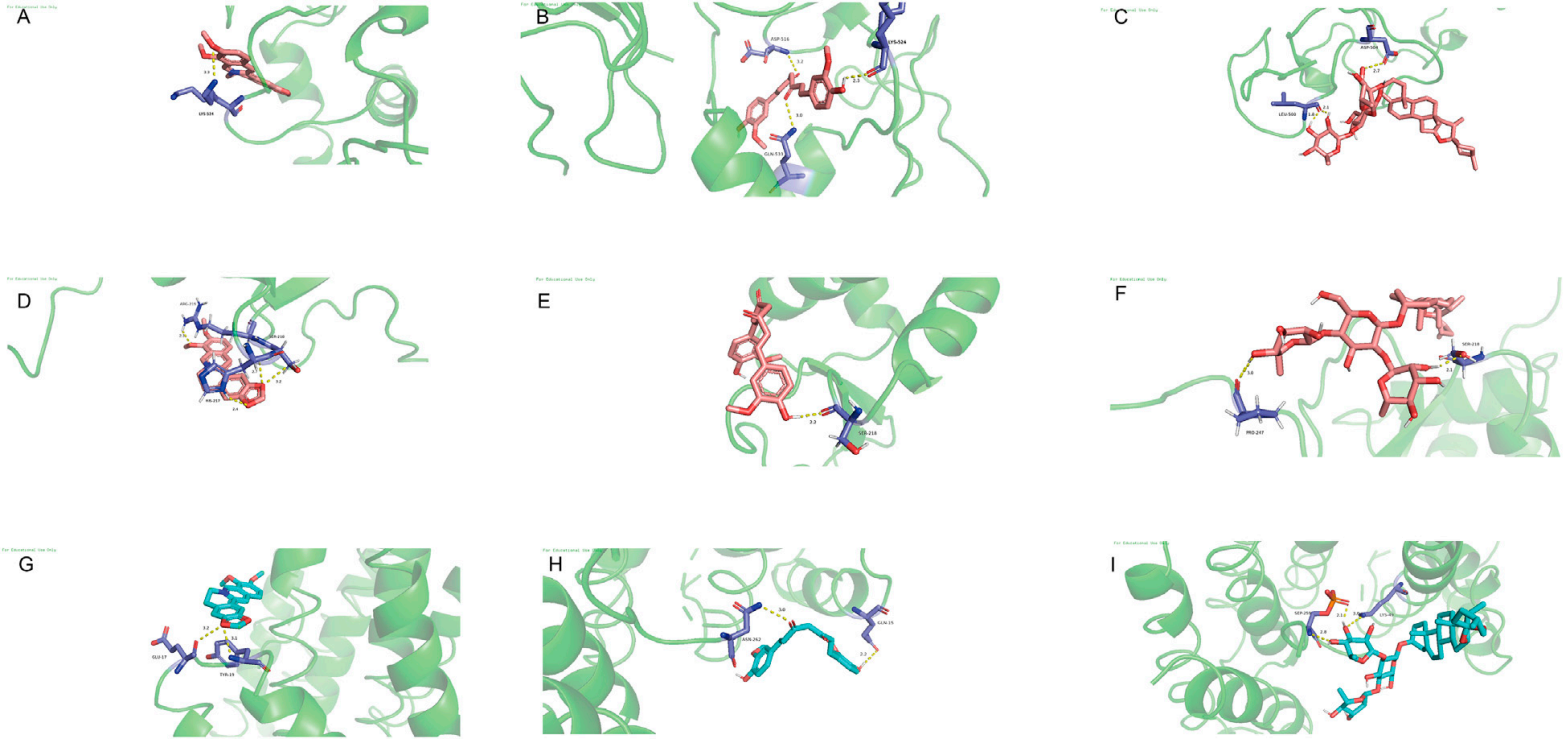
Molecular docking. **(A)** Curcumin with BRAF. **(B)** Curcumin with FOXO3. **(C)** Curcumin with RAF1. **(D)** berberine with BRAF. **(E)** berberine with FOXO3. **(F)** Berberine with RAF1. **(G)** Dioscin with BRAF. **(H)** Dioscin with FOXO3. **(I)** Dioscin with RAF1.

PyMOL 2.6.0 was used to prepare target proteins by removing water and small ligands, while AutoDockTools 1.5.7 was employed to add hydrogens to both target proteins and active molecules. The docking results were visualized with PyMOL (Figure 6). Binding energy below 0 indicates spontaneous binding between receptor and ligand, while values under −1.2 kcal/mol suggest robust docking and high stability. Our study found all binding energies were below −1.2 kcal/mol: Curcumin with BRAF (−6.53 kcal/mol), FOXO3 (−3.62 kcal/mol), RAF1 (−3.67 kcal/mol). Berberine with BRAF (−9.09 kcal/mol), FOXO3 (−4.21 kcal/mol), RAF1 (−4.83 kcal/mol). Dioscin with BRAF (−6.05 kcal/mol), FOXO3 (−2.38 kcal/mol), RAF1 (−2.87 kcal/mol). This indicates that these active compounds bind stably with core target proteins.

### 3.7 Histological analysis of the femoral head

**FIGURE 7:**
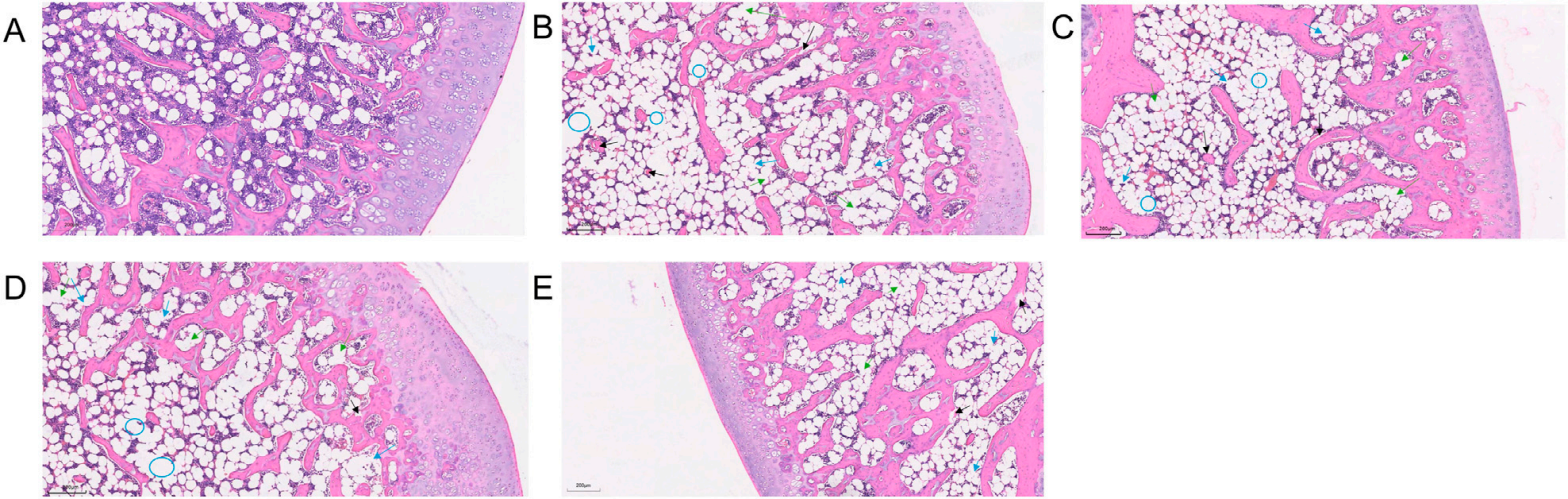
HE Staining Pattern. **(A)** Control group. **(B)** Model group. **(C)** Low-dose SBD group. **(D)** Medium-dose SBD group. **(E)** High-dose SBD group.

**FIGURE 8:**
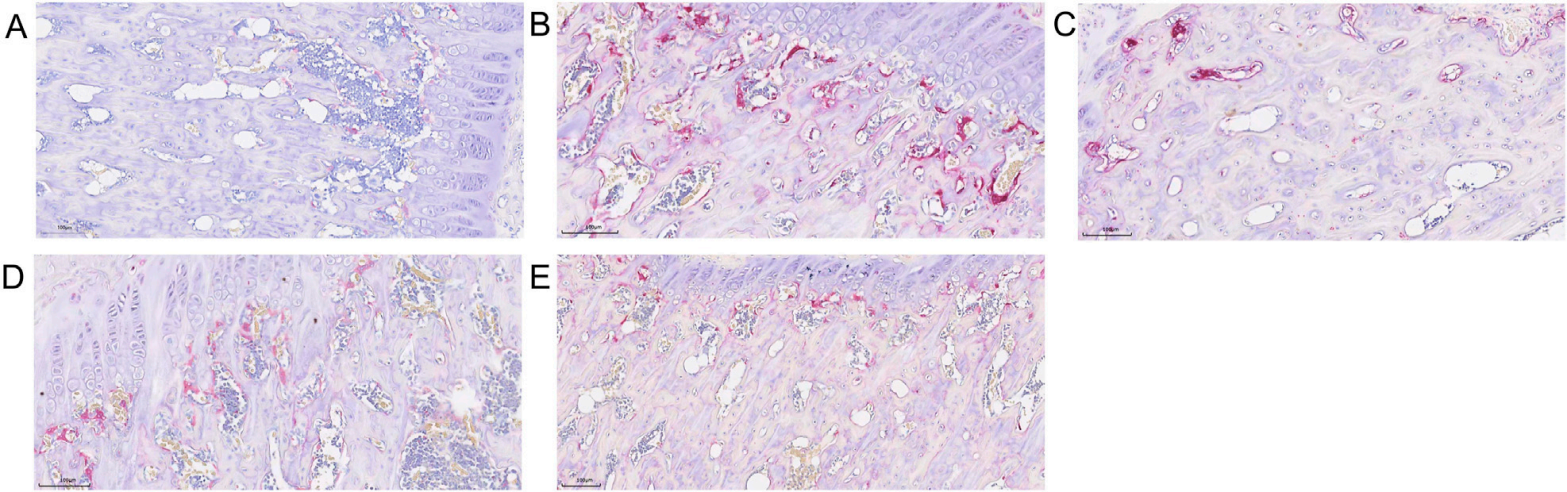
Trap Staining Graphic. **(A)** Control group. **(B)** Model group. **(C)** Low-dose SBD group. **(D)** Medium-dose SBD group. **(E)** High-dose SBD group.

Under HE staining, the control group’s articular cartilage displayed a uniform pink matrix with clearly stained blue nuclei, while unstained adipocytes appeared white. Cartilage thickness and cell number were normal, and trabeculae were closely arranged. Osteocytes were well-defined, featuring enlarged, evenly distributed chromatin, with no signs of apoptosis or empty lacunae. The marrow cavity contained abundant, morphologically normal cells, showing no abnormal proliferation or degeneration. Fat cells were sparse, and dense clusters of bone-forming cells beneath the trabeculae indicated active local bone formation (Figure 7A). In the model group, chondrocytes were disordered, and the cartilage matrix was unevenly distributed with some areas missing (black arrows). In necrotic regions, trabecular density markedly declined, and some trabeculae were fractured (blue arrows), indicating compromised bone integrity. As the condition progressed, trabeculae became further disrupted, with empty lacunae (blue circles) increasing to an empty lacuna rate of (86.79 ± 6.48)%. Osteocyte nuclei underwent pyknosis, and degenerative changes intensified. Marrow cell counts declined in necrotic areas, reflecting enhanced cell death and degradation. Meanwhile, adipose tissue proliferated significantly, with some fat cells merging into vacuole-like structures (green arrows), indicating abnormal lipid metabolism. No evidence of tissue proliferation or repair was observed in necrotic areas (Figure 7B). In the SBD treatment groups, both the low-dose (empty lacuna rate 56.54% ± 13.60%) and medium-dose (32.42% ± 7.68%) groups showed reduced empty lacunae compared to the model group (Figures 8C, D). In the high-dose group, the cartilage surface exhibited mild peeling and fragmentation (black arrows), yet chondrocyte alignment improved despite uneven and partially missing matrix. Within the necrotic regions, some trabeculae remained fractured (blue arrows), but the empty lacuna rate significantly decreased to (16.82 ± 7.66)%, substantially lower than in the model group. In summary, SBD treatment markedly alleviated the pathological alterations of articular cartilage and trabeculae, mitigating degenerative bone damage, with the high-dose group demonstrating particularly notable improvements.

### 3.8 Comparison of osteoclast numbers in femoral head across groups

Trap staining revealed that the blank control group exhibited fewer TRAP-positive osteoclasts (Figure 8A), while the model group showed a significant and dense increase in osteoclasts (Figure 8B). In the SBD low, medium, and high-dose groups, scattered osteoclasts were still observable, albeit in reduced numbers, with the high-dose group demonstrating a particularly pronounced inhibitory effect on osteoclast formation (Figure 8).

### 3.9 SBD inhibits apoptosis in femoral head tissue

**FIGURE 9:**
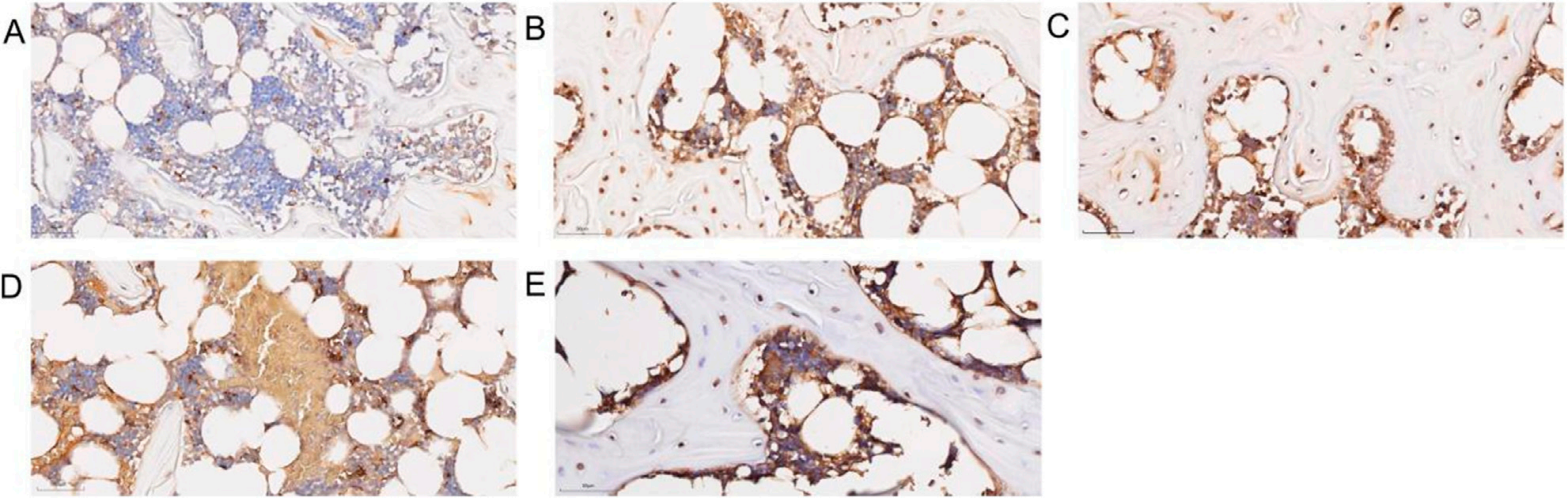
Diagram of apoptosis in bone tissue. **(A)** Control group. **(B)** Model group. **(C)** Low-dose SBD group. **(D)** Medium-dose SBD group. **(E)** High-dose SBD group.

Compared to the control group, the model group showed a significant increase in bone tissue cell apoptosis (Figure 9A). In contrast, the SBD intervention groups exhibited a notable reduction in apoptosis rates, which was dose-dependent. The high-dose group demonstrated a significantly lower apoptosis rate (Figure 9). This indicates that under these pathological conditions, SBD possesses anti-apoptotic properties.

### 3.10 SBD enhances vascular function

**FIGURE 10:**
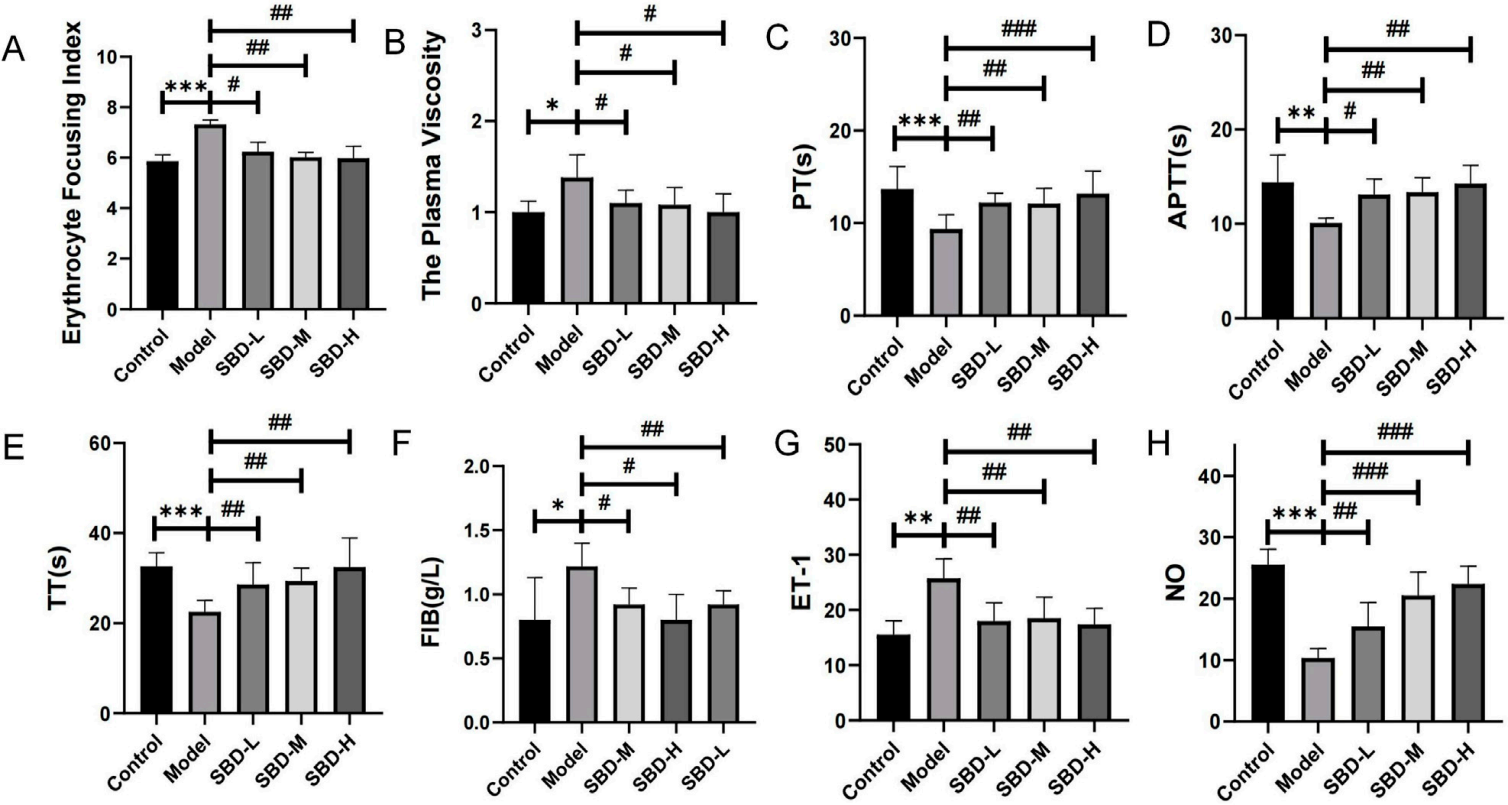
Comparison map of vascular function. (*p < 0.05, **p < 0.01, ***p < 0.001,#p < 0.05, ##p < 0.01, ###p < 0.001) **(A)** Erythrocyte Focusing Index: The model group exhibited a significant increase, while the treatment groups (SBD-L, SBD-M, SBD-H) demonstrated a marked reduction. **(B)** The Plasma Viscosity: The model group showed a significant elevation. SBD-L significantly reduced plasma viscosity, while SBD-L and SBD-H exhibited no notable change. **(C)** Prothrombin Time: The model group was significantly prolonged, and the treatment group was significantly shortened. SBD-H had the strongest effect in a dose-dependent manner. **(D)** Activated Partial Thromboplastin Time: The model group experienced a significant prolongation. All treatment groups significantly shortened PT, with SBD-H demonstrating the strongest effect. **(E)** Thrombin Time: The model group was significantly prolonged, and the treatment group was significantly shortened. The SBD-H effect was the most significant in a dose-dependent manner. **(F)** Fibrinogen: The model group displayed a significant increase, whereas SBD-L and SBD-M showed a notable decrease, with SBD-H exhibiting no significant change.**(G)** Endothelin-1: The model group demonstrated a significant increase, while the treatment groups showed a notable reduction, without displaying a clear dose-dependent relationship. **(H)** Nitric Oxide: The model group exhibited a significant decrease, whereas the treatment groups showed a marked increase, with SBD-H demonstrating the most pronounced effect, indicative of a dose-dependent nature.

Compared to the control group, the model group showed significantly increased hemorheological parameters, including blood viscosity and erythrocyte aggregation index. SBD treatment significantly reduced these parameters (Figures 10A, B). Coagulation indicators such as Prothrombin Time (PT), Activated Partial Thromboplastin Time (APTT), Thrombin Time (TT), and Fibrinogen (FIB) were assessed. The model group exhibited decreased TT, PT, and APTT levels and increased FIB compared to controls. SBD treatment improved TT, PT, and APTT levels and reduced FIB content, with statistical significance (*P* < 0.05). Higher doses of SBD showed more significant effects (Figures 10C–F). To evaluate SBD’s impact on vascular contraction and relaxation, serum levels of the vasoconstrictor Endothelin-1 (ET-1) and the vasodilator indicator Nitric Oxide (NO) were measured. The model group showed increased ET-1 and decreased NO compared to controls. SBD intervention significantly reduced ET-1 and increased NO, with statistical significance (*P* < 0.05) (Figures 10G, H).

### 3.11 SBD mitigates SONFH progression

**FIGURE 11:**
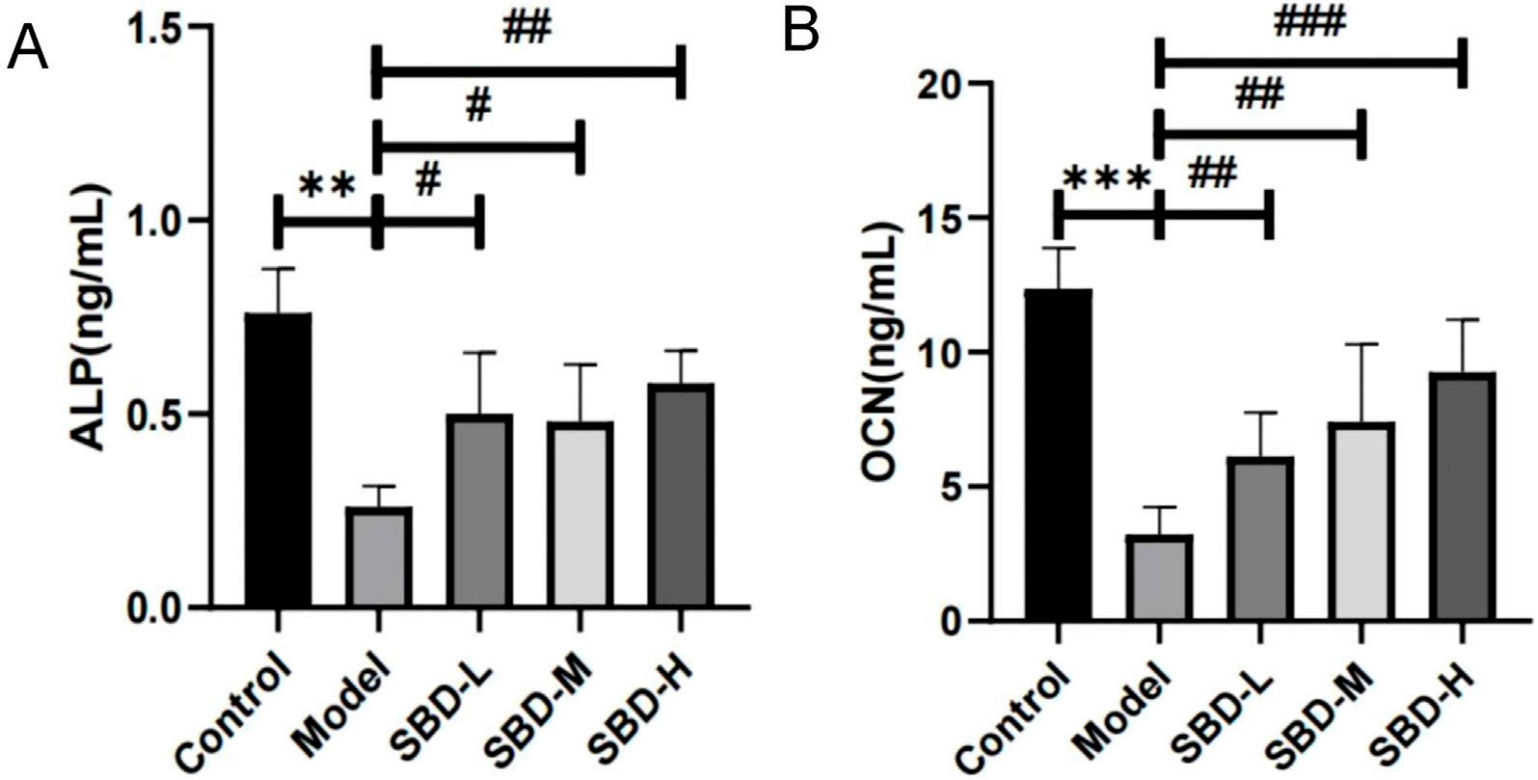
ELISA analysis of the effect of SBD on osteogenic markers in SONFH serum (**p < 0.01, ***p < 0.001,#p < 0.05, ##p < 0.01, ###p < 0.001). **(A)** ALP: The ALP level was highest in the Control group, significantly exceeding that of the Model group. The Model group exhibited a marked reduction in ALP levels, while the treatment groups showed a significant increase. **(B)** OCN: The OCN level was highest in the Control group, significantly surpassing that of the Model group, where OCN levels were markedly reduced. In contrast, the treatment groups exhibited a significant increase in OCN levels, demonstrating a clear dose-dependent relationship.

To further explore the impact of SBD on SONFH, we measured serum levels of ALP and OCN utilizing ELISA. Our findings indicate that SBD increases the levels of these osteogenic markers in SONFH serum (Figure 11).

### 3.12 SBD enhances osteoblast differentiation and mineralization

**FIGURE 12:**
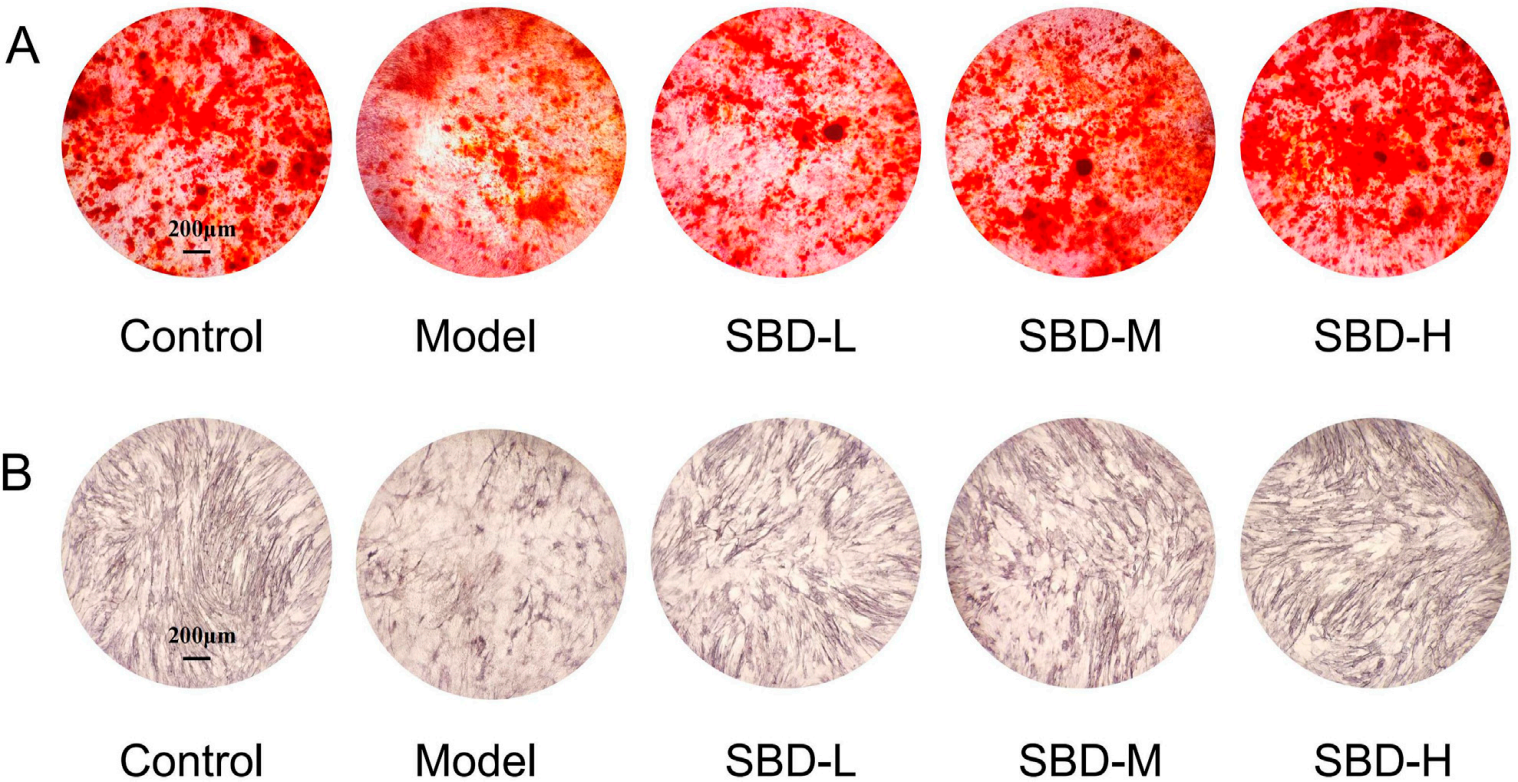
Effects of SBD intervention on ALP activity and mineralization in model cells. **(A)** ALP staining. **(B)** Alizarin Red staining.

The results of ALP staining demonstrated that ALP activity was suppressed in the model group compared to the control group. However, the SBD intervention groups exhibited increased ALP activity relative to the model group (Figure 12A). Cell mineralization was quantified using Alizarin Red staining. The findings indicated that cell mineralization was significantly reduced in the model group compared to the control group, whereas the SBD intervention groups showed enhanced cell mineralization (Figure 12B).

### 3.13 SBD activates VEGF and PI3K/Akt pathways to inhibit osteoblast apoptosis

**FIGURE 13:**
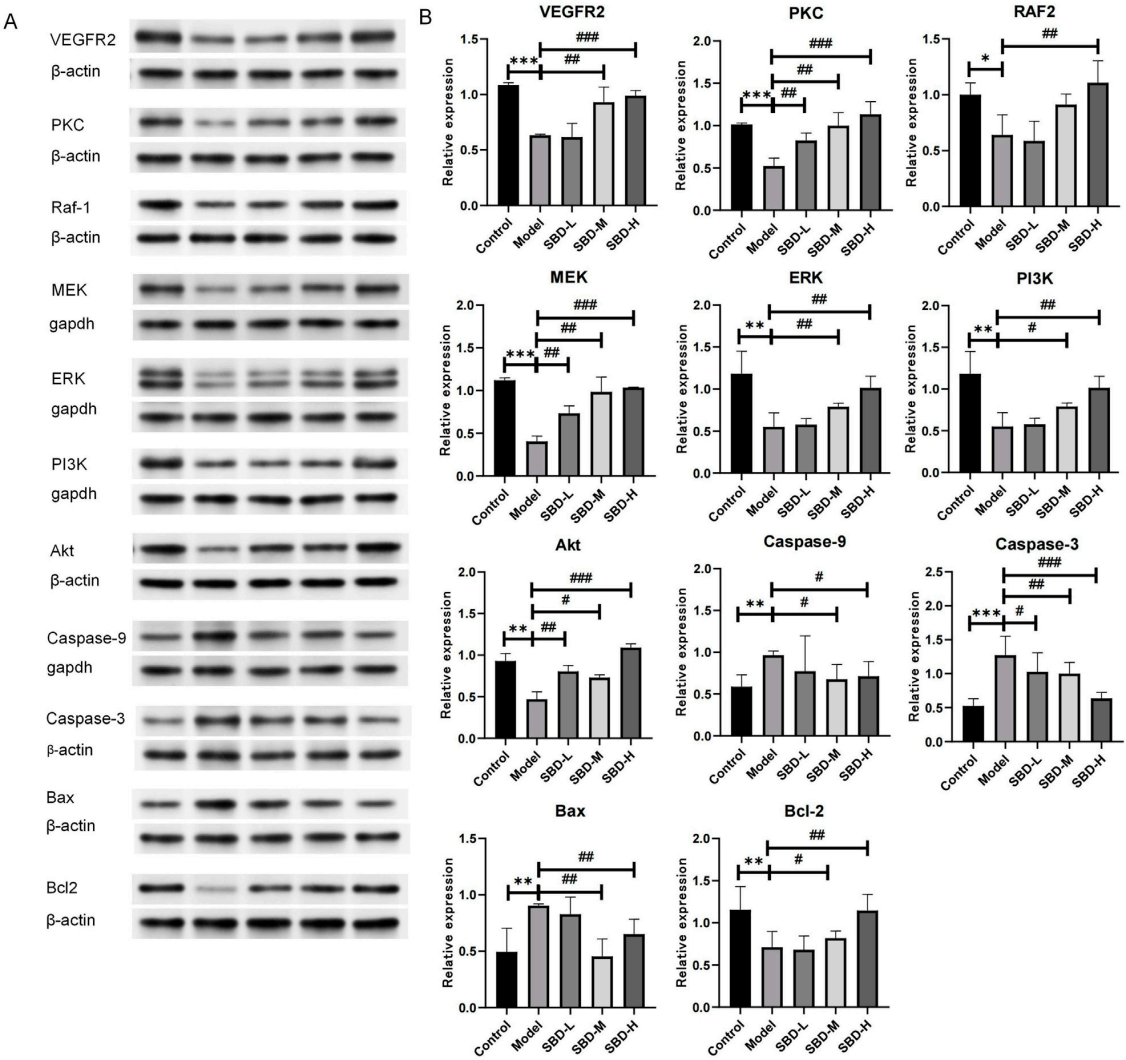
Validation of analysis results via Western blot. **(A)** The Western blot analysis revealed the expression profiles of VEGFR2, PKC, Raf-1, MEK, ERK, PI3K, Akt, Caspase-9, Caspase-3, Bax, and Bcl-2 proteins, with *β*-actin serving as the internal reference. **(B)** In the Model group, the expression of proteins VEGFR2, PKC, Raf-1, MEK, ERK, PI3K, Bxl-2 and Akt was significantly diminished. In contrast, the SBD-L, SBD-M, and SBD-H groups exhibited a notable increase in protein expression compared to the Model group. The expression of pro-apoptotic proteins (Caspase-9, Caspase-3, and Bax) was significantly elevated in the Model group. However, in the SBD-H group, the expression of Bax protein was unexpectedly increased, and the expression of other proteins was significantly decreased in the SBD-L, SBD-M and SBD-H groups.

To validate the enrichment analysis results, Western blot was employed to assess the PI3K/Akt and VEGF pathways as well as apoptosis-related targets. Compared to the control group, the model group exhibited suppressed expression levels of PI3K/Akt pathway targets (PI3K, Akt) and VEGF pathway targets (VEGFR2, PKC, RAF1, MEK, ERK). Following SBD intervention, these targets were upregulated in a dose-dependent manner, with the high-dose SBD showing more pronounced effects (Figure 13A). In the model group, the expression of Caspase-9, Caspase-3, and Bax was elevated, while Bcl-2 expression was reduced, indicating increased osteoblast apoptosis. SBD inhibited this apoptosis in a dose-dependent fashion (Figure 13B), suggesting that SBD can suppress osteoblast apoptosis.

## 4 Discussion

SONFH predominantly affects young and middle-aged individuals, with excessive or prolonged use of corticosteroids being the primary etiology, leading to femoral head collapse and hip damage (Liu et al., 2023). Corticosteroids not only regulate carbohydrate, lipid, and protein metabolism but also influence oxidative stress, cell proliferation, and apoptosis (Yu et al., 2015). They can accelerate osteonecrosis by promoting apoptosis of osteoblasts and osteocytes, ultimately disrupting bone microcirculation and inhibiting angiogenesis and the infiltration of new blood vessels into necrotic bone (Fan et al., 2014).

In recent years, natural products derived from TCM have garnered attention due to their safety and potential benefits, and have been utilized in the treatment of various bone diseases such as osteoporosis and ONFH. The proliferation, differentiation, and maturation of osteoblasts are crucial processes for bone formation and the maintenance of bone quality (Kim et al., 2014). SBD, originating from a clinically experienced formulation, has demonstrated promising efficacy against SONFH in preliminary observations. Combined network pharmacology analysis suggests that SBD may promote angiogenesis and inhibit osteoblast apoptosis by regulating the VEGF and PI3K/Akt pathways. Key active components include Curcumin, Berberine, and Dioscin, while FOXO3, RAF1, and BRAF serve as potential target links.

To enhance the success rate of clinical translation, this study employed a rat model due to the anatomical and physiological similarities of the femoral head to humans. Previous studies have demonstrated that excessive corticosteroid use induces endoplasmic reticulum stress and upregulates Bax expression, increasing the Bax/Bcl-2 ratio, activating the caspase cascade, and triggering osteoblast apoptosis and differentiation disorders (Almeida et al., 2007; Duan et al., 2020). Similarly, our findings demonstrated that, compared to the control group, DEX significantly disrupts bone tissue integrity and increases the rate of empty bone lacunae, indicating osteocyte loss. This was further confirmed by TUNEL assays, which showed a heightened apoptosis rate in the bone tissue of the model group. Additionally, TRAP staining revealed elevated levels of osteoclasts in the model group, suggesting that active osteoclastogenesis contributes to bone tissue degradation, a process supported by previous studies (Ren et al., 2023).

This study further explored the influence of SBD on the osteogenic process in SONFH, utilizing a combined approach of *in vivo* and *in vitro* experiments. The results revealed that SBD markedly enhanced the expression of osteoblast-associated proteins (such as OCN and ALP) within the femoral head tissues of SONFH rat models. CN, a hormone-like polypeptide synthesized and secreted by osteoblasts, and OCN, a biochemical marker of osteoblast activity, both signify an augmentation of osteoblast functionality (Berlier et al., 2019). Additionally, ALP activity serves as a sensitive indicator, reflecting the extent of osteoblast differentiation and their functional state (Li et al., 2019). Our research identified that prepared Rehmannia substantially elevated ALP activity (Xia et al., 2019a), aligning with our study’s outcomes. To further elucidate the underlying mechanisms of osteoblast involvement, we specifically extracted drug-containing serum and conducted *in vitro* assays. Following SBD intervention, the Bax/Bcl-2 ratio in osteoblasts was significantly reduced, and the expression of Caspase3 and Caspase9 was markedly inhibited, suggesting an improvement in osteoblast apoptosis. Concurrently, the model group exhibited an upregulation of the caspase protein family, particularly caspase-3, which was associated with osteoblast apoptosis and other cellular apoptotic processes. Caspase-3 plays a crucial role in osteoblast apoptosis associated with osteonecrosis, and its marked elevation is one of the characteristic pathological features of SONFH(Xu et al., 2014; Dai et al., 2017). Consequently, SBD may exert its anti-SONFH effects by inhibiting osteoblast apoptosis. Additionally, the assessment of osteoblast differentiation involves indicators such as ALP activity, collagen content, and mineralization levels (Cui et al., 2018). The study results indicate that DRGE significantly stimulates bone formation by enhancing ALP activity and promoting the formation of calcified nodules. Similarly, research has shown that Rehmannia glutinosa can enhance the proliferation, differentiation, and mineralization levels of osteoblasts treated with Dex (Xia et al., 2019b).

Furthermore, this study found that SBD ameliorated microcirculatory disturbances and blood rheological abnormalities induced by SONFH. In the SBD intervention groups, levels of TT, PT, and APTT were elevated, FIB levels were reduced, and blood fluidity was improved. Additionally, ET-1 levels decreased while NO levels increased, indicating reduced vascular resistance and enhanced microcirculation. These findings are consistent with the TCM concepts of “blood nourishing” and “marrow tonifying” to support bone health, and they provide modern medical evidence supporting the mechanisms by which traditional Chinese medicine treats SONFH.

VEGF is a crucial upstream signaling protein and an effective regulator of angiogenesis, playing a vital role in bone formation and repair associated with blood vessels (Gao et al., 2019). VEGFR2 primarily transduces molecular signals by regulating protein kinase C (PKC). Increased PKC activity can activate Raf kinase and endothelial nitric oxide synthase (NOS), leading to NO release (Jiao et al., 2019). The MEK/ERK signaling pathway, linked to endothelial cell motility, promotes angiogenesis by stimulating endothelial cell differentiation and migration. In this study, we observed significant downregulation of VEGFR2, PKC, Raf1, MEK, and ERK in rats and DEX-inhibited cells, a result consistent with findings by Yang et al. (Yang et al., 2023). Our results suggest that SBD effectively reactivates VEGF signaling suppressed by glucocorticoids, thereby promoting angiogenesis and improving vascular function, potentially via activation of the VEGFR2-PKC-Raf1-MEK-ERK pathway.

Previous research has indicated that activation of the PI3K/Akt pathway can prevent osteoblast apoptosis, as Akt reduces the risk of cell damage during oxidative stress, free radical exposure, and DNA damage (Lu et al., 2017). In addition, earlier studies have demonstrated that salidroside exerts anti-apoptotic effects on cardiac H9c2 cells, PC12 cells, and osteoblasts by activating the PI3K/Akt pathway (Xue et al., 2018). Therefore, we hypothesize that the anti-apoptotic effect of SBD may also be associated with PI3K/Akt pathway activation.

Subsequently, we identified three hub genes as promising therapeutic targets for SONFH after rigorous screening. FOXO3, a key member of the forkhead box transcription factor family, regulates diverse biological processes including apoptosis, oxidative stress responses, and metabolic regulation. Studies have shown that FOXO3 is targeted by miR-29a-3p, which inhibits FOXO3 expression and subsequently downregulates the Wnt/β-catenin signaling pathway, thereby reducing cell viability and osteogenic differentiation (Wang et al., 2022). Raf1, a critical node in the VEGF pathway, interacts with RAS proteins to activate downstream MEK and ERK, ultimately promoting angiogenesis. BRAF primarily regulates the MAPK/ERK signaling pathway, which is crucial for cell proliferation, differentiation, and survival. However, data regarding BRAF expression levels and changes in steroid-induced osteonecrosis of the femoral head remain limited. In this study, these hub genes exhibited differential expression in SONFH and demonstrated high predictive performance for disease onset. These findings highlight their potential as critical therapeutic targets of SBD and underscore unexplored opportunities for more effective SONFH treatments.

Due to the complexity of the disease mechanism of SONFH, effective intervention strategies remain scarce. This study unveils the potential role of traditional Chinese medicine, indicating its ability to stimulate angiogenesis through the promotion of VEGF and PI3K/Akt signaling pathways, and to inhibit osteoblast apoptosis, thereby enhancing osteoblast differentiation and mineralization, and safeguarding bone tissue cells from damage and structural degradation. The research suggests that this therapeutic effect may be mediated through targets such as FOXO3, RAF1, and BRAF, offering significant treatment benefits for patients with SONFH. These findings suggest that SBD offers a “multi-target, multi-mechanism” approach, enhancing its efficacy against SONFH and advancing the clinical application of traditional medicine. Additionally, it provides promising therapeutic targets and directions for SONFH treatment.

## Data Availability

The original contributions presented in the study are included in the article/Supplementary Material, further inquiries can be directed to the corresponding author.
